# A Neurophysiological Stratification Framework for Intermediate Motor Imagery-BCI Users Based on Independent Event-Related Brain Dynamics

**DOI:** 10.3390/brainsci16020202

**Published:** 2026-02-09

**Authors:** Xu Duan, Songyun Xie, Yujie Cui, Ting Ji, Hao Yan

**Affiliations:** 1 Key Laboratory for Artificial Intelligence and Cognitive Neuroscience of Language, Xi’an International Studies University, Xi’an 710128, China; xuerduan@xisu.edu.cn (X.D.);; 2 School of Artificial Intelligence, Northwestern Polytechnical University, Xi’an 710072, China

**Keywords:** brain-computer interface, motor imagery, BCI illiteracy, neurophysiological stratification, EEG characterization, independent component analysis

## Abstract

**Background:** Motor imagery-based brain-computer interfaces (MI-BCIs) enable individuals who are unable to perform physical movements to interact with the external world by imagining movements. Users are typically classified as good performers or BCI-illiterate based on the classification accuracy of distinct EEG patterns (e.g., 60% or 70%). Yet, studies show that approximately 70% of users fall within intermediate accuracies between 60% and 80%, and although exceed the chance level, they often fail to achieve reliable MI-BCI control. Intermediate users often exhibit asymmetric motor imagery abilities between left and right hands, highlighting the need for refined early assessment and stratified training approaches. **Methods:** We employed ICA to decompose each participant’s EEG data and extract independent ERD/ERS components as indicators using a rule-based automated framework. This framework integrated dipole localization, ERD/ERS characteristics, and frequency-band power features of ICs. Importantly, we applied a power spectral parameterization approach to remove the 1/f-like background activity in power estimation and used statistical methods to precisely estimate the latency and duration of ERD. The extracted indicators were subsequently subjected to clustering analysis to categorize participants into four groups. **Results:** In addition to good performers (24.8%) and poor performers (35.8%), two groups were identified: LgoodRpoor (27.5%), who performed well in left-hand MI but poorly in right-hand MI, and LpoorRgood (11.9%), who showed the opposite pattern. Notably, these unilateral performers did not show significant differences in contralateral ERD but exhibited substantial differences in ipsilateral ERS. **Conclusions:** The proposed independent event-related brain dynamics model enables more refined stratification of MI-BCI users. Findings from this characterization study may inform the design of graded training protocols, especially for users demonstrating unilateral motor imagery proficiency.

## 1. Introduction

Brain-computer interface (BCI) is an innovative control paradigm that operates independently of the human peripheral nerves and muscular activity [1,2]. Motor imagery (MI) refers to mental rehearsals of behaviors without any overt physical movement. Patients who are unable to perform a movement can create specific electroencephalogram (EEG) patterns to interact with the outside world simply by imagining body movement through MI-BCI [3,4]. To ensure reliable BCI control, users must learn to voluntarily produce brain activity patterns that are both distinguishable across different mental tasks and stable within a given task [5,6,7]. Unfortunately, a subset of individuals fails to generate sufficiently distinct modulation patterns. This phenomenon is commonly referred to as BCI illiteracy and represents a major obstacle to the broader applicability of MI-BCI [8].

Classification accuracy has been widely used to differentiate “good performers” from “BCI-illiterate” users [9]. The rates of BCI illiteracy vary widely, ranging from 6.7% [8], 15–30% [10] to over 50% [11], which is due in part to inconsistent threshold definitions of BCI illiteracy (e.g., 60% or 70% accuracy). A study reported that among 99 participants, 93% achieved classification accuracies above 60%, with 70% falling within the 60–80% range and only 20% exceeding 80% accuracy [8]. These findings indicate that the majority of users belonged to an intermediate performance group which, although surpassing the chance level, often failed to achieve precise control of MI-BCI systems. Notably, some individuals exhibited more pronounced neurophysiological features during left-hand motor imagery (LH-MI) and weaker features during right-hand motor imagery (RH-MI), while others showed the opposite pattern, with stronger EEG features during RH-MI and weaker EEG response during LH-MI [12,13].

Such unilateral advantages in EEG patterns lead to insufficient classification accuracies for reliable MI-BCI control. In EEG classification, left-hand and right-hand MI EEG signals are treated as equally important classes in covariance matrix-based discriminative learning [14,15]. If the neural feature between the two classes is imbalanced, it may introduce model bias, leading the classifier to preferentially learn from the stronger class and to misclassify signals from the weaker class [16,17,18]. Moreover, in asynchronous BCI systems, if the weaker MI signals are similar to the resting-state EEG, this can result in poorly defined decision boundaries [19]. Moreover, the classification accuracy metric is heavily dependent on model performance and does not directly reflect the underlying neurophysiological variability among users [20,21].

Event-related desynchronization (ERD)/synchronization (ERS) dynamics are widely recognized as reliable indicators of MI engagement. ERD and ERS reflect task-related decreases and increases in EEG power, respectively, and are key markers of cortical activation and deactivation. During both actual and imagined movements, ERD is typically observed in the mu and beta bands over contralateral motor areas, while ERS may occur in ipsilateral regions associated with movement cessation or attention shifts [22,23,24]. The mu rhythm (8–13 Hz), a sensorimotor EEG oscillation, is known to reflect motor-related cortical activity. Blankertz et al. (2010) estimated sensorimotor rhythm (SMR) by averaging mu and beta power over C3 and C4 [10]. Velasquez-Martinez et al. (2020) introduced an entropy-based method using vector quantization patterns to estimate ERD/ERS, achieving a moderate correlation with BCI performance (r = 0.53) [25]. Jorajuria et al. (2023) combined SMR power with phase synchronization in the mu and beta bands to improve evaluation accuracy [26]. Angulo-Sherman et al. (2025) further demonstrated that mu-ERD lateralization and the lateralized directed connectivity patterns in the mu and beta bands are closely linked to individual MI proficiency [27]. However, most existing studies rely on channel-level analyses. It remains confounded by volume conduction and background EEG activity, limiting the precision of mu/beta (de)synchronization measurements.

Independent component analysis (ICA) effectively mitigates volume conduction in EEG by isolating spatially distinct neural sources [28,29]. Researchers have proved that independent component-level analysis can substantially enhance the detectability, stability, and spatial consistency of movement-related activity [30]. The identification of MI-related ICs varies across studies. Duann et al. (2016) extracted topographies, time-frequency features, and dipole locations for each IC, using k-means clustering to group them into 15 clusters, with the SMR-ERD cluster selected via visual inspection [31]. Frolov et al. (2017) ranked ICs by their discriminative power across LH-MI, RH-MI, and rest, selecting the top three based on classification performance [32]. Melnik et al. (2017) used stimulus- and response-locked inter-trial coherence to categorize ICs into sensorimotor, sensory, motor, or unspecified groups [33]. Xu et al. (2019) proposed three criteria for identifying sensorimotor ICs: central scalp distribution, dipole localization in pre/postcentral gyri, and mu-band suppression [34].

Based on the above considerations, this study is guided by the following hypotheses:

**H1:**  *An ICA-based, refined framework could automatically detect both contralateral and ipsilateral MI-related ICs.*

**H2:**  *These indicators enable a quantitative explanation of MI-BCI performance.*

**H3:**  *Contralateral and ipsilateral ERD/ERS-related indices provide complementary information that enables a more comprehensive characterization of unilateral intermediate MI-BCI user profiles.*

To test these hypotheses, we propose an evaluation framework aimed at providing more accurate and interpretable stratification of individual MI-BCI proficiency, especially for the intermediate users. Our specific contributions are threefold:

(1) We introduce a rule-based automated ICA pipeline that identifies contralateral ERD-related and ipsilateral ERS-related independent components by integrating dipole localization, ERD/ERS significance patterns, and oscillatory peak characterization.
(2) We construct interpretable neurophysiological indices (e.g., contralateral ERD% and ipsilateral relative ERS power) to quantify individual MI proficiency beyond accuracy-based metrics.
(3) We demonstrate a finer-grained stratification of MI-BCI users into four profiles, including two unilateral intermediate groups, which provides implications for graded training design.

## 2. Materials and Methods

### 2.1. EEG Data Description

The PhysioNet database was used to evaluate the feasibility of the independent event-related brain dynamics framework for stratify individual variability in MI-BCI performance [35]. The EEG Motor Movement/Imagery Dataset v1.0.0 comprises over 1500 EEG recordings from 109 participants, collected using a 64-channel BCI2000 system. The signals were originally sampled at 160 Hz, and all data were re-referenced to the common average reference. Each participant completed 14 experimental runs, including two one-minute baseline runs and three two-minute runs for each of the following tasks: opening and closing the left or right fist, imagining opening and closing the left or right fist, opening and closing either both fists or both feet, and imagining opening and closing either both fists or both feet. These four tasks were performed sequentially and repeated twice. This study focused exclusively on the second motor imagery task. During this task, participants were instructed to imagine opening either their left or right fist in random order across 15 trials, with each imagery period lasting approximately 4.2 s, preceded by a rest period of approximately 4.2 s.

### 2.2. An Event-Related Brain Dynamics Framework Based on Independent Component Analysis

**Figure 1:**
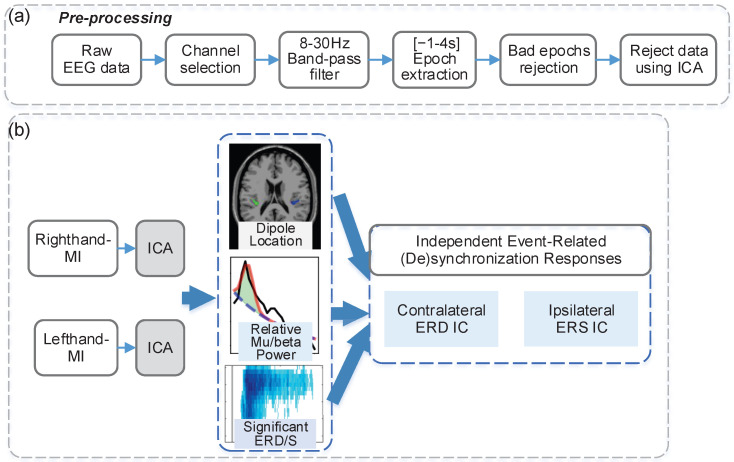
Flowchart illustrating the preprocessing, ICA decomposition, and selection of independent event-related (de)synchronization responses for motor imagery EEG data. (**a**) EEG preprocessing, (**b**) ICA decomposition pipeline and the selection rules for identifying contralateral ERD-related and ipsilateral ERS-related independent components based on dipole localization, relative mu/beta power, and statistically significant ERD/ERS patterns.

The event-related brain dynamics framework for motor imagery was constructed through four steps, as illustrated in Figure 1. First, the raw EEG data were preprocessed to obtain clean task-related EEG epochs. Next, the EEG epochs were categorized into two classes corresponding to left-hand and right-hand MI tasks, and ICA decomposition was performed separately for each class. Subsequently, for each IC, dipole source localization was performed based on its scalp topography, while time-frequency dynamics and relative mu/beta power were computed from its time series. In addition, we applied statistical methods for accurate estimation of the latency and duration of ERD, ensuring that observed ERD/ERS patterns reflect meaningful motor imagery effects rather than random noise. Finally, based on the established neuroimaging and electrophysiological evidence of motor imagery, such as spatial localization, temporal dynamics and frequency specificity, a set of rules was applied to automatically select the contralateral ERD-related IC and ipsilateral ERS-related IC for each task.

#### 2.2.1. EEG Preprocessing

The EEG preprocessing procedure is illustrated in Figure 1a. First, A subset of 30 channels (F3, Fz, F4, FT7, FC5, FC3, FC1, FCz, FC2, FC4, FC6, FT8, C5, C3, C1, Cz, C2, C4, C6, CP3, CP1, CP2, CP4, P5, P3, Pz, P4, P6, PO7, PO8) were selected, symmetrically distributed across the frontal lobe, central parietal lobe, and posterior parietal lobe. The selected EEG data were band-pass filtered between 8 Hz and 30 Hz to focus on oscillatory amplitude variations within mu and beta bands using zero-phase Hamming windowed sinc FIR filter. Subsequently, EEG epochs were extracted from 1 s before to 4 s after the task prompt for each trial across three experimental runs. Specifically, the 1-s segment preceding the task prompt served as the reference period, while the post-prompt segment constituted the task-related period. Artifact-contaminated epochs were rejected using “Reject data epochs” function in EEGLAB. Specifically, epochs were first screened using the abnormal values criterion, and any epoch containing signal amplitudes exceeding ±25 μV in any channel was marked for rejection. In addition, abnormal trends were identified using a maximum linear slope threshold of 50 μV per epoch with an R^2^ limit of 0.3, to detect epochs exhibiting excessive linear drifts. Statistical outliers were further detected using the improbable data and abnormal distributions criteria, with both single-channel and all-channel limits set to 5 standard deviations. Finally, abnormal spectral content was evaluated, and epochs with power spectra exceeding ±25 dB were marked as artifacts. After applying ICA to the preprocessed EEG data, the same rejection criteria were applied to the IC time series to further improve data quality. Each rejection step resulted in the removal of approximately 10% of the original epochs.

After preprocessing, an average of 85 valid trials per subject were retained (including both left-hand and right-hand motor imagery tasks), corresponding to a total analyzed duration of 382.5 s.

#### 2.2.2. ICA Decomposition

ICA provides a data-driven approach to separate statistically independent source processes and has been widely used to isolate sensorimotor-related neural components in EEG, and it is employed to obtain subject-specific, functionally interpretable components that enable more reliable characterization of ERD/ERS dynamics underlying motor imagery. In this study, we applied the Infomax ICA algorithm, which estimates a demixing matrix *W* based on the principle of information maximization, to recover underlying source signals from the recorded EEG data [36]. Infomax ICA was performed separately on the EEG data for the two different tasks for each participant, with the 30 selected channels decomposed into 30 independent components, using EEGLAB. All ICs were subjected to equivalent current dipole fitting using the DIPFIT2 plug-in. Components with a residual variance greater than 15% were excluded, as they are unlikely to originate from a single neural source [33]. Additionally, components exhibiting focal activation confined to a single electrode (e.g., muscle artifacts) were also removed. Following component selection, the power spectral density and time-frequency decomposition were computed for each IC.

#### 2.2.3. Relative mu/beta Power

In analyzing IC power spectrum, traditional narrow-band spectral analysis is prone to inadvertently include aperiodic activities outside the actual physiological oscillation band. We employed a spectral parameterization framework to identify oscillatory peaks after removing the 1/f-like component from the original power spectral density (PSD) [37]. FOOOF (Fitting Oscillations & One-Over-F) algorithm parameterizes the neural PSD into periodic and aperiodic components by identifying oscillations as spectral peaks exceeding the modeled aperiodic background, thereby providing relative narrow-band power estimates free from confounding aperiodic activity [38]. In FOOOF, the EEG power spectrum is modeled as:

(1) $$PSD=L+\sum_{n=0}^{N}G_{n}$$

where PSD is a combination of the aperiodic component *L* and $G_{n}$ (*N* Gaussian functions), with *N* is the total number of peaks extracted from the power spectrum. Each $G_{n}$ is a Gaussian function fitted to a peak, and is modeled as follows:

(2) $$G_{n}=a\cdot \exp\left(-\frac{{\left(F-c\right)}^{2}}{2w^{2}}\right)$$

where *a* denotes the peak’s amplitude, measured in $\log_{10}\left(power\right)$. In this study, if multiple $G_{n}$ components are present, the value of *a* corresponding to the component with the largest amplitude is referred to as the relative power. *c* is the center frequency. *w* is the standard deviation of the Gaussian function, and the two-sided *w* is defined as frequency width. *F* is the input frequency vector. The aperiodic component *L* is modeled across the entire spectral range using a Lorentzian function:

(3) $$L=b-\log(k+F^{\chi })$$

where *b* represents the broadband offset, χ is the spectral exponent, *k* controls the “knee” of the aperiodic curve. The peak’s frequency width is defined as the adjusted two-sided standard deviation of the Gaussian fit.

#### 2.2.4. Dynamic Power Changes and Significant ERD/ERS

The ERD and ERS phenomena induced by motor imagery tasks are time-locked but not phase-locked. Accordingly, ERD and ERS could be defined as the proportional power decrease and power increase relative to a reference interval, respectively [23]. We conducted time-frequency decomposition on the IC time series using the Morlet wavelet transform with a 3-cycle and Hanning window. ERD(t)% and ERS(t)% represent the time-resolved proportional changes in spectral power in the mu/beta band within the [−1, 4] s time window and is computed as follows:

(4) $$\mathrm{ERD}/S\left(t\right)\%=\frac{P(t)-R}{R}\times 100\%$$

where P(t) represents the power at time *t* within a specific frequency band, and *R* denotes the mean power in [−1, 0] s. We used the 1-s pre-prompt interval [−1, 0] s as the baseline to obtain a temporally proximal, task-unrelated reference.

(5) $$R=\frac{1}{r_{0}}\sum_{t=-1}^{0}P\left(t\right)$$

where $r_{0}$ is the number of samples within the reference interval. Notably, the scalar ERD% and ERS% values were defined as the average of ERD/ERS(t)% over the 0–4 s motor imagery period.

Next, we evaluated whether the time-frequency decomposition at each time point and frequency exhibited statistically significant differences relative to the reference interval. The pseudo-*T* statistics were computed by extensive random sampling between the reference region and each test point, generating a distribution of pseudo-*T* values and estimating the significance level at each resel [39]. False Discovery Rate correction was applied for multiple comparisons. For each significant resel, we assessed connectivity with neighboring resels in both horizontal (time) and vertical (frequency) directions, grouping adjacent resels into connected regions. The mean power change was calculated for each connected region. Regions with a mean value less than zero were classified as significant ERD regions, while those with a mean value greater than zero were classified as significant ERS regions. The area of each significant ERD/ERS region was quantified by the number of constituent resels, and latency was defined as the earliest time point among all significant resels. For each IC, the connected region with the largest area was selected as the representative significant ERD/ERS region.

#### 2.2.5. Selection Rule of Independent Event-Related (De)Synchronization Components

To identify the hand motor imagery-related ICs which show either contralateral ERD or ipsilateral ERS for each participant, the components extracted via ICA were automatically examined based on dipole source localization, relative mu/beta power, and significant ERD/ERS patterns, as illustrated in Figure 1b. The identification of contralateral ERD ICs followed three rules:

1. The source location must fall within the contralateral motor imagery-related brain regions, including the contralateral somatosensory cortex (BA1–3), primary motor cortex (BA4), superior parietal lobule (BA5, BA7), premotor and supplementary motor areas (BA6), anterior cingulate cortex (BA24, BA32), fusiform gyrus body-selective area (BA37), inferior parietal lobule (BA40), opercular regions (BA44, BA45), and middle frontal gyrus (BA9, BA46) [40,41,42,43].
2. Among IC candidates satisfying Rule 1, the top three components with the highest relative power were selected.
3. From these candidates, the IC with the largest area of 4-connected regions in the significant ERD map was chosen. If none of the candidates exhibited significant ERD, the IC with the highest relative power was selected.

The identification of ipsilateral ERS ICs was based on similar criteria:

1. The source location must be within ipsilateral motor imagery-related brain regions (same as Rule 1 for ERD ICs).
2. The relative power spectrum must exhibit prominent oscillatory peaks within the mu/beta frequency range.
3. The IC with the largest area of 4-connected regions in the significant ERS map was selected. If none of the candidates exhibited significant ERS, the IC with the highest relative mu/beta power was chosen.

### 2.3. Regression Model

To validate the effectiveness of the proposed neurophysiological model and assess the contribution of selected IC features to individual MI-BCI performance, we extracted eight-dimensional feature vectors from four ICs for each participant. Rather than using complex machine learning models, a multiple linear regression was deliberately employed to preserve interpretability and to quantify the independent contribution of each neurophysiological indicator. The extracted features included ERD%, relative ERD power, ERS%, and relative ERS power during both LH-MI and RH-MI. These features were then used as independent variables to construct a multiple linear regression model, with MI-BCI performance scores serving as the dependent variable. In addition to explanatory power, robustness was also assessed. A 10-fold cross-validation procedure was additionally performed. In each fold, the model was trained on 90% of the participants and evaluated on the remaining 10%.

To quantify MI-BCI performance, we adopted the widely used metric of class distinctiveness [5]. The distinctiveness between two EEG patterns was measured using the Fisher criterion adapted to the Riemannian geometry of covariance matrices:

(6) $$ClassDis\left(A,B\right)=\frac{\delta_{R}\left(\bar{P_{A}},\bar{P_{B}}\right)}{\frac{1}{2}\left(\sigma_{P_{A}}+\sigma_{P_{B}}\right)}$$

where $\bar{P_{A}}$ and $\bar{P_{B}}$ denote the Riemannian means, and $\sigma_{P_{A}}$ and $\sigma_{P_{B}}$ represent the mean absolute deviation of EEG covariance matrices for class *A* and class *B*, respectively. $\delta_{R}$ is the Riemannian distance between the class means. A higher ClassDis value indicates greater inter-class separation relative to intra-class variability, reflecting better MI-BCI performance.

In addition to the dynamic power change and relative power features, several additional metrics were considered, including the area and latency of significant ERD/ERS, and the center frequency of the spectral peak. Pearson correlation analyses were conducted to examine the relationships between these metrics and MI-BCI performance.

### 2.4. Clustering Analysis

We employed clustering analysis based on significant indicators associated with left- and right-hand MI performance to stratify participants into finer-grained subgroups. Specifically, ERD%, relative ERS power during LH-MI, and ClassDis were selected as input features for k-means clustering (k=2) [44]. Participants with higher ClassDis values were classified as good performers in LH-MI, while those with lower ClassDis values were classified as poor performers in LH-MI. The same procedure was applied to cluster participants according to the performance of right-hand MI based on ERD%, relative ERS power during RH-MI, and ClassDis. Based on the clustering results, participants who performed well in both LH-MI and RH-MI were labeled as “good performers”. Those who performed well in left-hand but poorly in right-hand MI were labeled as “LgoodRpoor”; those with the opposite pattern were labeled as “LpoorRgood”; and participants with poor performance in both tasks were labeled as “poor performers”.

Subsequently, we analyzed differences across the four clustered groups using one-way ANOVA followed by Tukey-Kramer’s HSD post hoc tests for each of the four neurophysiological features. For the relative ERS power during LH-MI, the assumption of homogeneity of variances was violated; therefore, Welch’s ANOVA was employed along with Games-Howell post hoc tests.

## 3. Results

Section 3.1 focuses on mechanistic characterization of independent ERD/ERS components, Section 3.2 provides a quantitative explanation of MI-BCI performance through regression modeling, and Section 3.3 emphasizes practical user stratification based on performance-relevant features.

### 3.1. Selection of Contralateral ERD and Ipsilateral ERS ICs

This section aims to examine whether the proposed ICA-based rules can reliably capture both contralateral and ipsilateral neural responses during left-hand and right-hand motor imagery tasks. The validity of the extracted independent components is evaluated from three complementary perspectives: dipole source localization, spectral features, and statistically significant time–frequency patterns.

#### 3.1.1. Dipole Source Localization of MI-Related ICs

**Figure 2:**
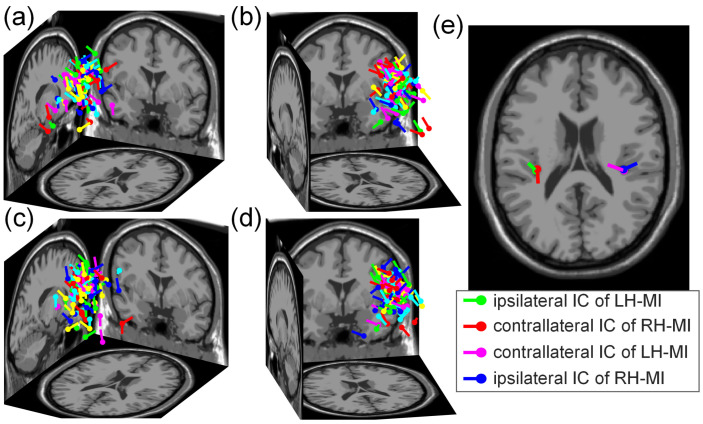
Dipole source localization of independent event-related (de)synchronization responses. Panels (**a**–**d**) show the distribution of dipole source locations for the selected independent components (ICs) associated with ipsilateral ERS and contralateral ERD during left-hand (LH-MI) and right-hand motor imagery (RH-MI) tasks, respectively. Specifically, (**a**) ipsilateral ICs of LH-MI, (**b**) contralateral ICs of LH-MI, (**c**) contralateral ICs of RH-MI, and (**d**) ipsilateral ICs of RH-MI. Panel (**e**) shows the average dipole source locations across 109 participants for the ipsilateral and contralateral ICs during LH-MI and RH-MI.

Following the MI-related IC selection criteria, contralateral and ipsilateral ICs corresponding to left- and right-hand MI were successfully identified for each participant. The scalp topography of contralateral ERD IC and the ipsilateral ERS IC for both LH-MI and RH-MI for the first 48 participants are illustrated in Table S1. Figure 2a–d display the dipole source localizations of 109 participants for the ipsilateral IC during LH-MI, the contralateral IC during LH-MI, the contralateral IC during RH-MI, and the ipsilateral IC during RH-MI, respectively. The results demonstrate that the dipole sources of these four ICs were consistently localized within motor-related brain regions across participants. Figure 2e shows the average dipole source locations for the four selected ICs across all 109 participants. The mean MNI coordinates for the ipsilateral IC during LH-MI and the contralateral IC during RH-MI were [−34.98, −28.48, 44.85] (postcentral gyrus, BA1–3) and [−33.81, −26.37, 44.89] (premotor cortex, BA6), respectively. For the contralateral IC during LH-MI and the ipsilateral IC during RH-MI, the mean MNI coordinates were [36.32, −26.14, 44.10] and [36.67, −26.99, 43.87], both located within the postcentral gyrus (BA1–3).

#### 3.1.2. Spectral Features of Contralateral and Ipsilateral ICs

**Figure 3:**
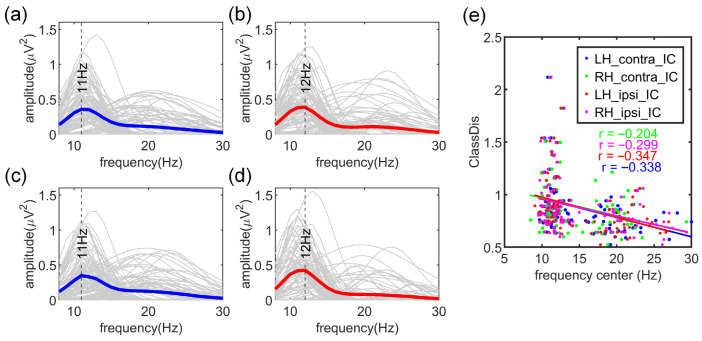
Power spectral density (PSD) of the dominant oscillatory components for the selected ICs: (**a**) contralateral ICs in LH-MI, (**b**) ipsilateral ICs in LH-MI, (**c**) contralateral ICs in RH-MI, and (**d**) ipsilateral ICs in RH-MI. The bold blue and red lines represent the average PSDs across 109 participants for ERD and ERS ICs, respectively. (**e**) Relationship between the center frequency of the dominant peak and ClassDis for the four IC types. Colored lines indicate linear regression fits for each group.

Figure 3a–d illustrates the modeling of oscillatory neural activity using a Gaussian-based approach, where each IC is characterized by the relative power, frequency bandwidth, and center frequency of its dominant periodic components. The average center frequency of contralateral ICs during LH-MI and RH-MI tasks exhibited prominent peaks at 11 Hz (lower mu band), whereas the average center frequency of ipsilateral ICs during both tasks peaks at 12 Hz (upper mu band). Furthermore, the center frequencies of both contralateral and ipsilateral ICs for left- and right-hand MI were negatively correlated with ClassDis ($r_{\mathrm{LH-ERD}}=-0.338,\;p_{\mathrm{LH-ERD}}<0.001;\;r_{\mathrm{LH-ERS}}=-0.347,\;$$p_{\mathrm{LH-ERS}}<0.001;\;$$r_{\mathrm{RH-ERD}}=-0.204,\;p_{\mathrm{RH-ERD}}=0.041;\;r_{\mathrm{RH-ERS}}=-0.299,\;p_{\mathrm{RH-ERS}}=0.003$) (Figure 3e).

#### 3.1.3. Significant Time-Frequency Patterns of MI-Related ICs

**Figure 4:**
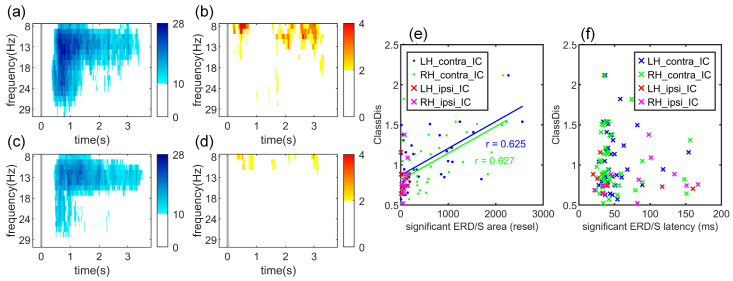
Time-frequency maps of significant ERD/ERS for the selected ICs: (**a**) contralateral ICs in LH-MI, (**b**) ipsilateral ICs in LH-MI, (**c**) contralateral ICs in RH-MI, and (**d**) ipsilateral ICs in RH-MI. Blue and red colors indicate the number of occurrences of significant ERD and ERS, respectively. Panels (**e**,**f**) show the relationships between ClassDis and (**e**) the area of significant ERD/ERS and (**f**) the latency of significant ERD/ERS for the four selected ICs. Lines represent linear regression fits for each group.

Figure 4a–d presents color-coded maps showing the number of participants exhibiting significant ERD/ERS at each resel. Darker regions indicate resels where significant ERD/ERS was observed in a greater number of participants, whereas white regions represent resels where no significant ERD/ERS was observed. The color scale was adjusted separately for different conditions to facilitate comparison between ERD and ERS results. The results show that significant ERD during both left- and right-hand MI primarily occurred within the mu band, typically spanning the entire imagery period with a concentration in the early phase of the task. The average latencies of significant ERD for left- and right-hand MI were 470 ms and 407 ms, respectively. Compared to ERD, significant ERS was observed much less frequently and covered a substantially smaller area. ERS responses were mainly confined to the mu band, exhibited considerable inter-subject variability in latency and duration, and typically did not persist for the full task period.

As shown in Figure 4e, the area of significant ERD (in resels) for contralateral IC during both left- and right-hand MI was positively correlated with ClassDis ($r_{\mathrm{LH}}=0.625,\;$$p_{\mathrm{LH}}<0.001;\;$$r_{\mathrm{RH}}=0.627,\;p_{\mathrm{RH}}<0.001$), and no significant correlation was found between ipsilateral IC during both tasks and ClassDis. Moreover, no statistically significant correlation was found between the latency of significant ERD/ERS and ClassDis for either hand (Figure 4f).

Therefore, this section provides reproducible contralateral ERD and ipsilateral ERS ICs, demonstrating the correlations between the frequency characteristics of the ICs and the area of significant ERD with performance, supporting the subsequent use of these IC features to explain ClassDis.

### 3.2. Results of the Regression Model

This section constitutes the primary quantitative evidence of the functional relevance between MI-related ICs and ClassDis. The *F*-test indicated that the multiple linear regression model was statistically significant (F(8,109)=22.676, p<0.001), with an adjusted $R^{2}$ of 0.645, demonstrating that the independent variables collectively explained 64.5% of the variance in the dependent variable. The cross-validated results yielded a mean absolute error (MAE) of 0.132 and a root mean square error (RMSE) of 0.167 across folds, indicating stable model performance across different subject subsets. The regression equation is expressed as:

(7) $$ClassDis=C+\sum_{i=1}^{8}k_{i}x_{i}$$

where $x_{1}$–$x_{4}$ represent ERD% of LH-MI, ERS% of LH-MI, ERD% of RH-MI, and ERS% of RH-MI, respectively; and $x_{5}$–$x_{8}$ correspond to the relative ERD power for LH-MI, relative ERS power for LH-MI, relative ERD power for RH-MI, and relative ERS power for RH-MI. The regression coefficients indicated that $x_{1},x_{3},x_{6}$ and $x_{8}$ made statistically significant contributions to ClassDis, as shown in Table 1.

### 3.3. User Performance Stratification

This section focuses on the practical stratification of MI-BCI users based on performance-relevant neurophysiological features, rather than further characterizing neural mechanism. We first present the performance stratification results obtained using clustering, and then describe the neural signatures of each group. The clustering analysis revealed that 27 participants (24.8% of the total) were identified as good performers. The LgoodRpoor group included 30 participants (27.5%), while the LpoorRgood group consisted of 13 participants (11.9%). The remaining 39 participants (35.8%) were classified as poor performers. Specifically, the ClassDis values were highest in the good group (M = 1.307, SD = 0.288), lowest in the poor group (M = 0.760, SD = 0.103), and intermediate in the unilateral groups (LgoodRpoor: M = 0.929, SD = 0.062; LpoorRgood: M = 0.923, SD = 0.122). This ordering demonstrates a consistent directional trend with MI-BCI performance.

**Figure 5:**
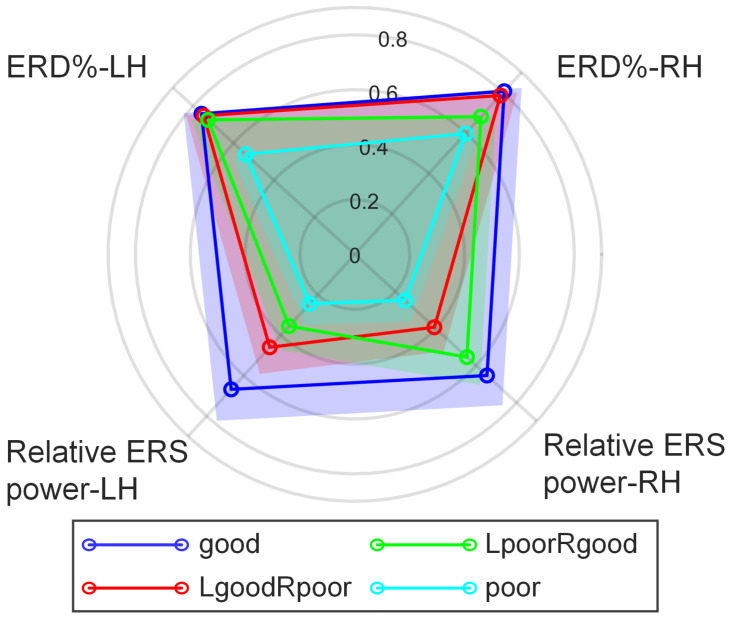
Radar chart illustrating the categorization of participants based on MI-BCI performance. Participants were clustered into four groups—good, LgoodRpoor, LpoorRgood, and poor—according to four characteristics: ERD% in LH-MI, ERD% in RH-MI, relative ERS power in LH-MI, and relative ERS power in RH-MI.

As shown in Figure 5, a radar plot was used to visualize the performance of the four clustered groups across four significant features. ERD% values were min-max normalized to the range [1, 0], with stronger (more negative) ERD% values mapped closer to 1 and weaker (more positive) ERD% values mapped closer to 0. The values of relative ERS power were normalized to the range [0, 1]. The radar plot clearly illustrated that good performers exhibited consistently high values across all four dimensions, indicating robust ERD% and relative ERS power responses. In contrast, poor performers showed the lowest values across all dimensions. The LgoodRpoor group displayed higher relative ERS power during left-hand MI and lower values during right-hand MI, whereas the LpoorRgood group showed the opposite pattern, with higher relative ERS power during right-hand and lower values during left-hand MI.

**Figure 6:**
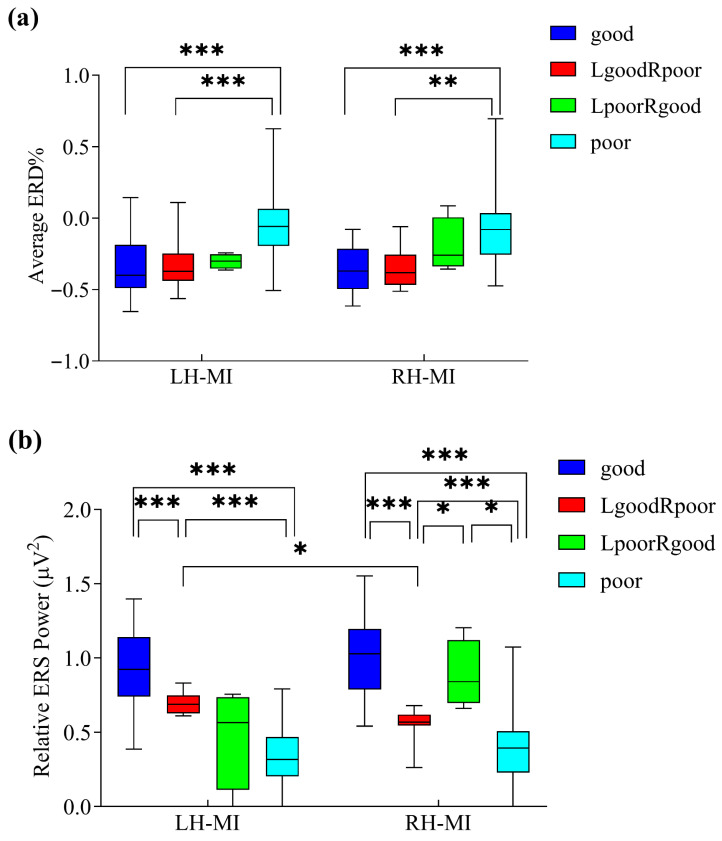
Group differences in contralateral ERD% and ipsilateral relative ERS power across participant groups. (**a**) Boxplots of contralateral ERD% during LH-MI and RH-MI tasks. (**b**) Boxplots of ipsilateral relative ERS power during LH-MI and RH-MI. Asterisks denote significant group differences (* p<0.05, ** p<0.01, *** p<0.001).

Figure 6a shows that both the good and LgoodRpoor groups exhibited significantly stronger ERD% during both LH-MI and RH-MI compared to the poor group (the statistical results are summarized in Table 2). Although the LpoorRgood group also showed stronger ERD% than the poor group during both LH-MI and RH-MI, the differences were not statistically significant.

As shown in Figure 6b, relative ERS power for both LH-MI and RH-MI exhibited a clear hierarchical trend across the four groups. Specifically, the good group demonstrated significantly higher relative ERS power than both the LgoodRpoor and poor groups, and the LgoodRpoor group showed significantly higher relative ERS power than the poor group in both LH-MI and RH-MI (the statistical results are summarized in Table 2). A shared characteristic of the LgoodRpoor and LpoorRgood groups was the absence of significant differences in ERD% between LH-MI and RH-MI. The newly defined LgoodRpoor and LpoorRgood groups revealed distinctive lateralized patterns in MI performance. Within the LgoodRpoor group, the relative ERS power during LH-MI was significantly higher than that during RH-MI (t=3.574, p=0.005). In LpoorRgood group, the relative ERS power during RH-MI was higher than that during LH-MI, reaching marginal significance (t=2.429, p=0.093). Moreover, during RH-MI, the relative ERS power in the LpoorRgood group was significantly higher than that in the LgoodRpoor group and the poor group. Both the LgoodRpoor and LpoorRgood groups showed no significant difference in ERD% between LH-MI and RH-MI. These findings suggest that for participants with unilateral MI dominance (i.e., LgoodRpoor and LpoorRgood), there is a clear advantage in ipsilateral relative ERS power, whereas no significant advantage is observed in contralateral mu/beta suppression.

Among the multiple analyses conducted, the regression model provides the primary quantitative explanation of MI-BCI performance, while clustering serves as an application-oriented step that translates these explanatory features into actionable user stratification.

## 4. Discussion

To investigate electrophysiological indicators underlying individual variability in MI-BCI performance, we proposed an independent event-related brain dynamics framework. We decomposed EEG signals to automatically identify independent neural sources associated with LH-MI and RH-MI, and to extract predictive features through regression model. Then, clustering participants based on these features revealed distinct neural profiles associated with bilateral or unilateral MI proficiency. Key findings indicated that (i) contralateral ERD% and ipsilateral relative ERS power are the primary indicators of MI-BCI performance; (ii) stronger MI performance is associated with larger significant ERD areas and mu-band dominance; and (iii) participants who only performed well in unilateral MI tasks typically did not exhibit significant differences in contralateral ERD, while exhibiting significant differences in ipsilateral ERS. In the following discussion, we evaluate the effectiveness of the proposed method in characterizing individual MI-BCI performance and analyze the participant performance categorization.

Given the limited spatial resolution of EEG, we identified left- and right-hand MI-related ICs on a broader spatial scale, guided by neuroimaging evidence. Results showed that the dipole sources of both ipsilateral and contralateral ICs during LH-MI and RH-MI were consistently localized within the posterior parietal cortex and the premotor cortex. The posterior parietal cortex, overlapping with the primary somatosensory cortex (BA1–3), is implicated in the reinstatement of sensorimotor representations and forms part of the sensorimotor loop with the primary motor cortex [40,42,43,45]. Mu-rhythm desynchronization during hand MI was predominantly generated in this region [32]. The premotor cortex (BA6) is central to motor planning, preparation, and rhythmic control during imagined movement [41,46].

Traditional neural spectral analyses based on predefined frequency bands may conflate oscillatory activity with non-oscillatory noise. To address this, we employed the FOOOF algorithm to isolate oscillatory peaks by removing the aperiodic component. Results showed that ipsilateral ERS exhibited a prominent peak in the upper mu band, comparable in magnitude to contralateral ERD, with its peak located in the lower mu range. This finding aligns with Pfurtscheller et al. (2006), who identified the lower mu as somatotopically unspecific and typically reflected as ERD, whereas upper mu is more somatotopically specific and often manifests as ERS [23]. Furthermore, individuals whose dominant peak was located in the mu band performed better than those with peaks in the beta band, suggesting that stronger MI performance is associated with more pronounced mu-ERD [47].

Significant ERD spanned a substantially larger area in the time-frequency spectrogram than the barely detectable ERS in both LH-MI and RH-MI, and the area of significant ERD was strongly correlated with MI performance. The average latency of significant ERD was 470 ms for LH-MI and 407 ms for RH-MI, indicating an earlier mu suppression compared with that reported in previous studies. For example, Duann et al. (2016) reported peak mu suppression at 635 ms [31]; Xu et al. (2019) observed half-max suppression at 550 ms with full ERD lasting 600–700 ms [34]; and Tidare et al. (2021) found peak ERD between 500–700 ms [48]. The earlier latencies identified in this paper suggest that the selection method of MI-related IC enabled more precise detection of contralateral ERD sources. However, the latency of significant ERD showed no relationship with MI-performance.

In conclusion, on the one hand, the MI-related IC identified through the proposed framework reliably captured clear contralateral ERD and ipsilateral ERS patterns across participants. On the other hand, as indicated by the regression model, the features extracted from these ICs - specifically ERD%, relative ERS power - explained 64.5% of the variance in MI-BCI performance. Together, these findings support the validity of the selection criteria and provide a reliable basis for understanding individual differences in MI-BCI performance.

Notably, the ICA decomposition and dipole fitting are intended as an offline, subject-specific calibration step rather than being recomputed in real time. After calibration, the learned unmixing matrix and the selected ERD/ERS-related independent components can be fixed. During online operation, incoming EEG segments are projected through the fixed unmixing matrix to obtain IC time series, and ERD/ERS indices are computed on the preselected ICs. Therefore, the online stage involves only linear projection and band-limited power estimation, which is computationally lightweight.

ANOVA results showed that good performers elicited contralateral ERD for both LH-MI and RH-MI, whereas poor performers did not show such desynchronization. Ipsilateral ERS exhibited a clear hierarchical pattern across four groups. Good performers demonstrated consistently strong ipsilateral ERS. Participants who performed well in LH-MI exhibited strong ipsilateral ERS in the left hemisphere, while those who performed well in RH-MI exhibited strong ipsilateral ERS in the right hemisphere. Poor performers, however, failed to elicit ipsilateral ERS during both LH-MI and RH-MI. Thus, ipsilateral ERS was identified as a key distinguishing feature for unilateral MI task performance. These findings suggest that contralateral ERD underpins general MI ability and serves as the neurophysiological basis for successful LH-MI and RH-MI tasks, while ipsilateral ERS serves as a critical distinguishing marker of performance variation, especially for LgoodRpoor and LpoorRgood groups.

Failure to elicit a clear ERD impairs the ability to control an MI-based BCI system. In contrast, ERS is a less readily evoked but more selective feature. When ipsilateral ERS is successfully induced, it consistently enhances MI performance, regardless of its latency or duration. This pattern reflects mechanisms of cortical inhibition and attentional resource allocation. One explanation involves inter-regional competition. The activation of a specific cortical area (left-hand motor cortex) may be accompanied by suppression in surrounding or functionally opposing regions (right-hand motor cortex) [49]. Another explanation relates to attentional shifts. When attention transitions from one effector (left-hand MI in a previous trial) to another (right-hand MI in the next trial), the SMR in the previously engaged contralateral region may rebound. This phenomenon is known as post-movement beta ERS, reflecting the reduced corticospinal excitability [50]. Compared to ERD, ERS likely involves enhanced attentional control and specific neuromodulatory mechanisms. As a result, ERS shows greater inter-individual variability than the more robust and widely observed contralateral ERD. Only a subset of individuals reliably exhibit ipsilateral ERS, whereas most show relatively weak ipsilateral ERD responses [51].

In addition, more individuals achieved better performance in LH-MI (27.5%) than in RH-MI (11.9%). Although the public dataset lacked handedness information, it is reasonable to assume that most participants were right-handed, based on the general population distribution. In right-handed individuals, the cortical regions responsible for left-hand movement are less frequently engaged, which requires greater recruitment of the right motor cortex during left-hand imagery. This increased cortical activation induced stronger mu-ERD, thereby enhancing the ability to generate mu-ERD during MI with the non-dominant (left) hand [52,53].

Based on the feature analysis of the four groups, we proposed a graded training protocol to enhance MI-BCI performance across different user types. For good performers, no additional training is required, as they could generate robust ERD/ERS patterns and effectively control MI-BCI systems. For participants categorized as LgoodRpoor and LpoorRgood, targeted unilateral MI training should be recommended to improve MI performance on the weaker side. As indicated by the earlier findings, training protocols should incorporate real-time feedback based on ipsilateral mu power, particularly relative ERS strength, to reinforce neural engagement in the underperforming hand. In contrast, poor performers may require more intensive and more prolonged training to elicit meaningful ERD/ERS responses. For this group, alternative BCI approaches such as affective-state-based BCIs [54] or visually evoked potential-based paradigms [55] may provide more reliable control pathways.

There are several limitations to this study:

1. Although the present study was conducted on a well-established and curated public dataset, our primary goal was not to address the full spectrum of challenges encountered in real-world MI-BCI scenarios, but rather to establish a clear and interpretable link between neurophysiological features and MI-BCI performance.
2. The present study intentionally focuses on event-related, component-level dynamics to achieve interpretable and performance-relevant characterization of MI-BCI users. We acknowledge that this component-level approach does not explicitly model network-level interactions or inter-regional connectivity, which may provide additional insight into distributed neural mechanisms underlying MI performance. Notably, independent components inherently integrate distributed neural sources, and event-related ERD/ERS has long been established as a gold-standard neural marker in MI-BCI studies. By comparison, connectivity-based measures are more difficult to translate into performance stratification and feedback-oriented applications Future work may extend the proposed framework by integrating connectivity-based measures or network-level modeling to further characterize distributed motor imagery networks under more challenging experimental conditions.
3. The present framework identifies neurophysiological heterogeneity among MI-BCI users, its ability to guide training requires prospective validation. A rigorous validation would involve a longitudinal, controlled study design in which participants are first stratified using the proposed IC-level ERD/ERS features, followed by group-specific training interventions. Baseline conditions could include non-stratified training, conventional accuracy-based grouping, or randomly assigned training protocols. Implementing such validation would require extending the current pipeline to support real-time feedback based on ipsilateral mu power. These components fall beyond the scope of the present study. Importantly, the current study does not claim superiority over existing training protocols, but rather provides a neurophysiological basis upon which baseline methods can be compared in future work.

## 5. Conclusions

This study proposed an independent event-related brain dynamics framework and identified contralateral ERD% and ipsilateral relative ERS power as key neurophysiological markers for assessing individual MI-BCI proficiency. Through clustering analysis, in addition to the good and poor performer groups, two newly defined groups, LgoodRpoor and LpoorRgood, were discovered, which exhibited asymmetrical motor imagery abilities between the left and right hand. By incorporating a real-time feedback protocol informed by ipsilateral relative ERS power, the proposed training enabled targeted reinforcement of lateralized deficiencies within motor imagery tasks. However, the effectiveness of the training protocol has not yet been rigorously validated. In future work, we plan to conduct an empirical study to confirm whether targeting ipsilateral ERS can effectively improve MI-BCI skills in users with unilateral MI deficiencies. Nevertheless, as the proposed training protocol remain descriptive at this stage and require rigorous validation to establish their practical effectiveness in MI-BCI training.

## Figures and Tables

**Table 1:** Summary of the explanatory linear regression analysis relating neurophysiological indicators to MI-BCI performance (ClassDis).

Predictor	LH-MI		RH-MI	
	β	***p***-Value	β	***p***-Value
ERD%	−0.307 **	0.002	−0.248 *	0.011
ERS%	0.161	0.165	0.153	0.135
Relative ERD power	−0.035	0.750	0.133	0.128
Relative ERS power	0.186 *	0.045	0.266 **	0.002

* *p* < 0.05, ** *p* < 0.01.

**Table 2:** Between-group comparisons of ERD% and relative ERS power.

Metric	MI Condition	Comparison	t-Value	*p*-Value
ERD%	LH-MI	good vs. poor	6.162 ***	<0.001
		LgoodRpoor vs. poor	4.358 ***	<0.001
	RH-MI	good vs. poor	6.409 ***	<0.001
		LgoodRpoor vs. poor	4.629 **	0.002
Relative ERS power	LH-MI	good vs. LgoodRpoor	4.543 ***	<0.001
		good vs. poor	11.284 ***	<0.001
		LgoodRpoor vs. poor	12.329 ***	<0.001
	RH-MI	good vs. LgoodRpoor	7.953 ***	<0.001
		good vs. poor	11.831 ***	<0.001
		LgoodRpoor vs. poor	4.161 ***	<0.001
		LpoorRgood vs. LgoodRpoor	4.261 ***	<0.001
		LpoorRgood vs. poor	7.532 ***	<0.001

** *p* < 0.01, *** *p* < 0.001.

## Data Availability

The datasets analysed during the current study are available in the [PhysioNet-EEG Motor Movement/Imagery Dataset] repository, https://www.physionet.org/content/eegmmidb/1.0.0/ (accessed on 5 April 2023).

## Supplementary Materials

The following supporting information can be downloaded at: https://www.mdpi.com/article/10.3390/brainsci16020202/s1, Table S1: The scalp topography of contralateral ERD IC and the ipsilateral ERS IC for both LH-MI and RH-MI for the first 48 participants.
